# Biodiversity and Community Analysis of Plant-Parasitic and Free-Living Nematodes Associated with Maize and Other Rotational Crops from Punjab, Pakistan

**DOI:** 10.3390/life11121426

**Published:** 2021-12-17

**Authors:** Aatika Sikandar, Tabassum Ara Khanum, Yuanyuan Wang

**Affiliations:** 1 National Nematological Research Centre, University of Karachi, Karachi 75270, Pakistan; aatika_sikander@yahoo.com (A.S.); tabassumak@uok.edu.pk (T.A.K.); 2 College of Biosciences and Biotechnology, Shenyang Agricultural University, Shenyang 110866, China

**Keywords:** maize, plant-parasitic nematodes, free-living nematodes, community analysis, cluster analysis, Punjab, Pakistan

## Abstract

Maize (*Zea mays* L.) is one of Pakistan’s essential staple food crops. Plant-parasitic nematodes (PPNs) are a significant restraint in maize production. However, free-living nematodes (FLNs) provide crucial ecological functions such as suppressing pests and nutrient mineralization. This study aimed to assess the community analysis of plant-parasitic and free-living nematodes associated with maize and other rotational crops (those cultivated in sequence with the maize in the same field) from Punjab, Pakistan. The occurrence percentage was observed per 500 g soil for each nematode genus. The present study revealed that 24 species of plant-parasitic and free-living nematodes were identified from maize crops and other rotational crops from 16 localities through Punjab, Pakistan. Nematode communities were analyzed by absolute frequency, relative frequency, relative density, and prominence value, while cluster analysis was based on the presence or absence of nematode in different localities. The overall proportion of plant-parasitic nematodes was 35%, while free-living soil nematodes recovered 65%, out of 210 samples of maize and other rotational crops. Several major genera of plant-parasitic nematodes were reported during the present study viz., *Ditylenchus*, *Filenchus, Helicotylenchus*, *Hemicriconemoides*, *Heterodera, Hoplolaimus, Malenchus, Pratylenchus*, *Psilenchus, Rotylenchulus, Seinura*, *Telotylenchus*, *Tylenchorhynchus*, and *Xiphinema* Community relationship revealed the overall dominance of *Heterodera zeae*, with the highest incidence (55.71%) followed by *Tylenchorhynchus elegans* (33.33%) and *Helicotylenchus certus* (24.76%). The results provide valuable information on the community structure of nematodes in maize and other rotational crops of maize in Punjab, Pakistan. Moreover, this data can be used as a preventive measure before PPN incidence results in greater losses on maize.

## 1. Introduction

Maize (*Zea mays* L.) is one of the important cereal crops grown worldwide [1]. It is the most preferred and popular crop in semi-arid and arid regions of the world [2]. It is a good source of food consumed throughout the world and an important feed component for livestock [3]. Maize is high-yielding, easy to digest, and cheaper than any other cereals [4]. Every part of this plant has an economic value: grain, cob, stalk, leaves, and tassel are used for producing a variety of food and non-food products [5]. Maize is a main source of starch (72%), proteins (10%), fiber (5.8%), vitamins A and B (3–5%), sugar (3.0%), oil (4.8%), and ash (1.7%) [6]. Moreover, 100 g of fresh grains contains 361 calories of energy, 74.4 g carbohydrate, 9.4 g protein, 4.3 g fat, 1.8 g fibres, 1.3 g ash, 9 mg calcium, 290 mg phosphorus, 2.5 mg iron, and 140 mg vitamin [7]. Maize is rich in starch, so it is used as raw material for several products, i.e., glucose, lactic acid, alcohol, plastic, starch, rayon, shoe polish, synthetic rubber, dextrose, sorbitol, dextrin, high fructose syrup, maltodextrin, gum, and artificial leather [8,9].

Punjab is the second largest Province of Pakistan, consisting of about 205,344 km^2^ (79.284 sq.ml.). Punjab has different soils, such as loam, sandy, clay, saline, river soil, so all crops are easily grown here [10,11]. In Pakistan, maize is the third important cereal after wheat and rice [12]. Maize accounts for 4.8% of the total cropped area and 3.5% of the value of the agricultural output of Pakistan [13]. Maize cultivation is mainly concentrated in 2 geographical areas, the 11 districts of Khyber Pakhtunkhwa and the 12 districts of Punjab [14]. Spring maize cultivation in Punjab has significantly increased since the positive involvement of multinationals in Pakistan [15]. During the spring, progressive farmers in Punjab yield more than 2.375 tones/acre and earn millions [16]. Maize is a crop of significant economic importance, but unfortunately, it suffers from several diseases caused by bacteria, fungi, insects, viruses, and nematodes [17,18].

Phytonematodes (Plant-parasitic nematodes) damaged the crops by feeding and formed the interaction with other organisms that pose a severe threat to agriculture throughout the world [19]. However, damage caused by nematodes is often difficult to distinguish from other factors due to their microscopic size [20]. Plant-parasitic nematodes are among the most important nematodes that live in soil, leaves, and mainly in roots [21,22]. Phytonematodes pose a significant threat to agriculture throughout the world; an estimated annual loss reached up to the U.S. $157 billion [23]. However, no such study was conducted in Pakistan on the percentage of crop losses due to plant-parasitic nematodes in maize. Some nematodes are migratory, while others are sedentary [24]. Moreover, most plant-parasitic nematodes feed on roots but some feed on foliar tissues of plants [25].

Several nematodes are associated with maize, but major ones belong to genera; *Meloidogyne, Heterodera, Pratylenchus, Helicotylenchus, Tylenchorhynchus, and Ditylenchus* [26]. *Heterodera zeae* is a significant pest of maize, commonly known as “maize cyst nematode” [27]. The soil, temperature, and other Punjab biotic and abiotic conditions are suitable for reproducing *H. zeae* [28]. Root-knot nematode belongs to the genus *Meloidogyne,* which is an economically important nematode of maize [29]. The crop market value and yield are reduced because they form extensive root galling in the plant [30]. Talwana et al. [31] and Adegbite [32] reported that the *Meloidogyne* species severely infected the maize crop. Lesion nematode *Pratylenchus zeae* considerably damaged the maize [33], and they formed the lesions in the plant, which facilitated other parasitic fungi, bacteria, and viruses; those caused secondary infection in the plant [34]. *Rotylenchulus reniformis is* also a severe pest of maize and other oilseed crops and pulses [35]. *Tylenchorhynchus zeae* exhibiting poor growth of maize crops [36]. *Helicotylenchus nannus* and *H. erythrinae* were associated with maize [37]. *Ditylenchus dipsaci* also considerably damaged the maize field [38].

In Pakistan, nematodes have been recognized as one of the limiting factors in agriculture production. There is a need to improve agricultural production. There is no recently ample evidence available in Pakistan on maize nematodes, especially about its major pest *H. zeae*, because no such study was performed after Maqbool and Shahina [28]. Thus, the surveillance and survey of maize and other crops cultivated in sequence with the maize in the same field were carried out to address this issue. In this regard, the study was conducted for the survey, prevalence, and population density of new and known nematode species. Therefore, this study will be a base for more research on the estimation of crop yield losses in maize and other crops cultivated with maize. In the future, this study will also divert the researchers’ attention toward the management of nematodes in maize in Pakistan.

## 2. Materials and Methods

### 2.1. Survey

**Figure 1:**
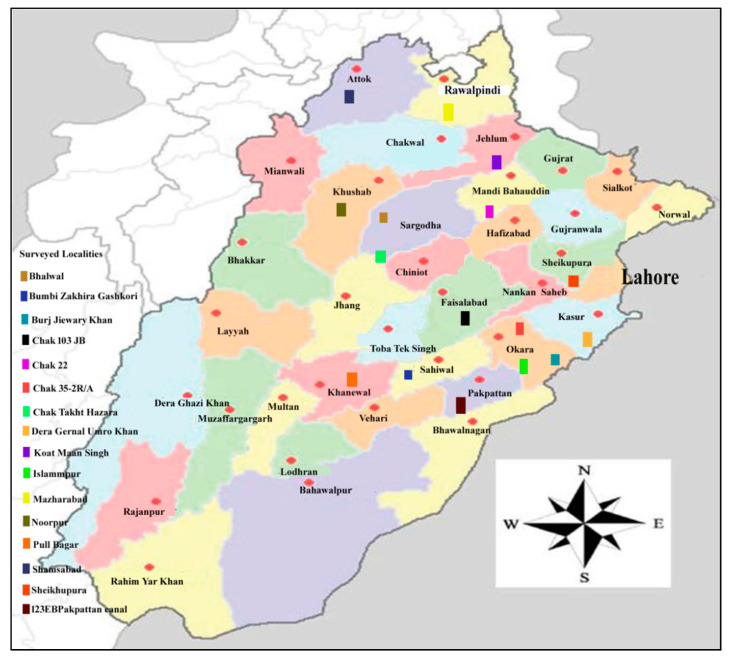
The map displayed survey sides in Punjab, Pakistan.

The current survey was performed at 16 locations in Punjab, Pakistan. Bhalwal, Bumbi Zakhira Gashkori, Burj Jieway Khan, Chak 103 JB, Chak 22, Chak 35-2R/A, Chak Takht Hazara, Dera General Umro Khan, Islampur, Koat Maan Singh, Mazaharabad, Noorpur, Pull Bagar, Shamsabad, Sheikhupura, and 123 EB Pakpattan Canal were the locations surveyed (Figure 1). A total of 210 soil and roots samples of maize and other rotational crops (potato, sorghum, wheat, tobacco, and green chilli) were obtained. The samples were collected during the spring season (March–April). Nematode distribution is rarely uniform or steady, and changes can happen quickly. The distribution of nematodes is usually uneven. Thus, the survey field that will be sampled was divided into subdivisions. Then, the effective sampling map was constructed. Large and small survey fields were sampled in a systematic, zigzag way. The main aim of the survey was to observe the prevalence and occurrence of nematode-related diseases.

### 2.2. Sampling

Root and soil samples were obtained to classify the distribution of nematodes while their higher populations were present [39]. Samples were collected at a depth of 10–15 cm with the aid of a hand shovel. Each sample contained 500 g of soil. The samples were stored in polythene bags and covered with rubber bands. All samples were appropriately marked with a permanent marker on the location, such as date, host, locality, and any other related data, which later helped classify the samples’ source.

### 2.3. Extraction of Cyst Nematodes and Eelworm Nematodes

The soil sample was approximately 500 g mixed, thoroughly stirred with water in a large bucket. Then, the suspension was left for 2–3 min to the deposition of soil particles. The suspension was passed through the 36 and then through 100 sieve mesh. The presence of cyst nematodes and several other long nematodes were deposited on these two sieves and were poured into a beaker. As described by Van Bezooijen [40], cyst extraction and counting were performed. For the extraction of these nematodes, the rest of the water contained an eel-shaped worm or nematodes; water passed through a 350-sieve mesh. Then, the suspension of 350 sieve mesh nematodes was spilled over a wet tissue paper mounted in a funnel so that water reached its bottom. The funnel was kept straight for 48 h to ensure the maximum population of nematodes. The 100 mL of nematode-containing water was drawn into a beaker after 48 h and analyzed under a stereoscopic binocular microscope [41].

### 2.4. Extraction of Root-Knot Nematodes

The roots were removed from the samples and sliced into 1–2 cm pieces. The roots were placed in a water-containing Petri dish. Every root piece was observed under the binocular microscope. Egg masses and females of root-knot nematodes (RKN) were removed by dissecting needles. Females were taken out cautiously, avoiding damage to their bodies. The collected females were moved to the cavity block with a dropper for further procedures, such as recognizing the species of *Meloidogyne* based on the perianal pattern. Root-knot nematode egg masses were transported to the cavity because larvae emerge from them, and their measurement helped to identify the species [40].

### 2.5. Identification of Nematodes

Standard morphological features were used in nematode identification. Different keys were used for the identification of nematodes at the species level [20,42]. For the higher categories and synonymy, Siddiqi’s [43] book was followed for the order Tylenchida. Hunt’s [44] classification was followed for the order Aphelenchida. The nomenclature provided by Jairajpuri and Ahmad [45] for the order Dorylaimida was used. Ahmad and Jairajpuri [46] nomenclature for the taxa of Mononchida was accepted for higher categories and synonyms in present details. However, Andrássy [47] and Andrássy’s [48] classification was followed forRabditida. Moreover, to further confirm species, the life cycles of nematodes were observed in the greenhouse in pots.

### 2.6. Data Analysis

#### 2.6.1. Community Analysis of Nematodes

According to Norton [49], community analysis of nematodes was calculated from 16 localities in Punjab, Pakistan. Parameters such as Absolute frequency (*AF*%), Relative frequency (*RF*%), Relative density (*RD*%), and Prominence value (*PV*) were used to describe the nematode community structure.

(1) $$Absolute\;frequency\;\%=\frac{Number\;of\;samples\;containing\;a\;species}{total\;number\;of\;samples\;examined}\times 100$$

(2) $$Relative\;frequency\;\%=\frac{Frequency\;\;of\;\;the\;\;species}{Sum\;of\;frequencies\;of\;all\;species}\times 100$$

(3) $$Relative\;density\;\%=\frac{Density\;\;of\;\;the\;\;species}{Sum\;of\;mean\;density\;of\;all\;nematode\;species\;}\times 100$$

(4) $$Prominence\;value=Density\times \sqrt{Frequency}$$

#### 2.6.2. Cluster Analysis

Cluster analysis of localities and nematodes, data based on the quantitative analysis (presence (1)/absence (0) of nematodes), which used to establish the similarity between localities based on Jaccard’s coefficient of similarity. The dendrograms constructed based on data were related to the localities. All of the computations were conducted using the MINITAB (version 18) computer application.

#### 2.6.3. Statistical Analysis

The diversity of nematodes was analyzed by one-way analysis of variance (ANOVA). The mean difference was calculated using Duncan’s multiple range test (*p* > 0.05) for the significance test. All of the statistical processes were administered by different statistical packages such as IBM-SPSS statistics 25.0 version software and Microsoft Excel. Graphs were constructed through Sigma Plot 10.0 software.

## 3. Results

### 3.1. Percentage Occurrence of Nematodes in Punjab, Pakistan

**Figure 2:**
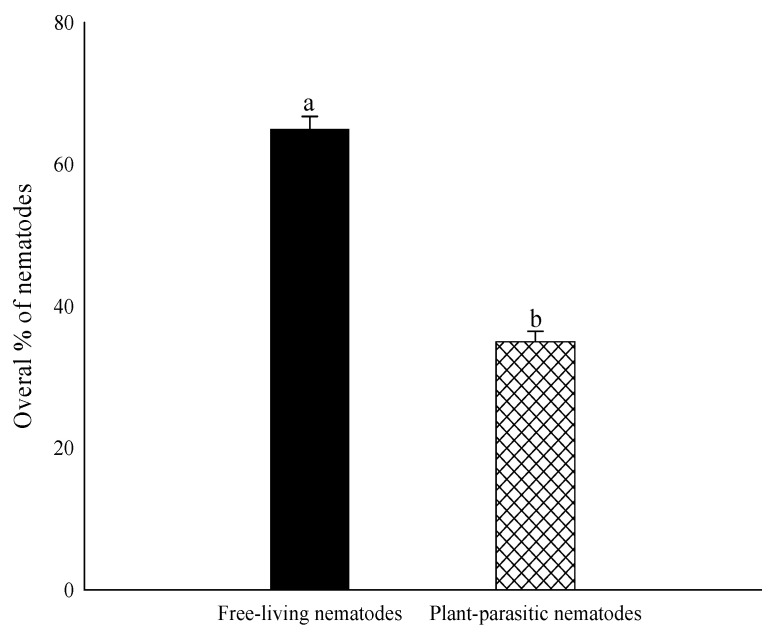
Occurrence (%) of plant-parasitic and free-living soil nematodes in maize and other rotational crops. The bars illustrated the mean and standard error. Different letters on the bars indicate significantly different values according to Duncan’s multiple range test at *p* > 0.05.

**Figure 3:**
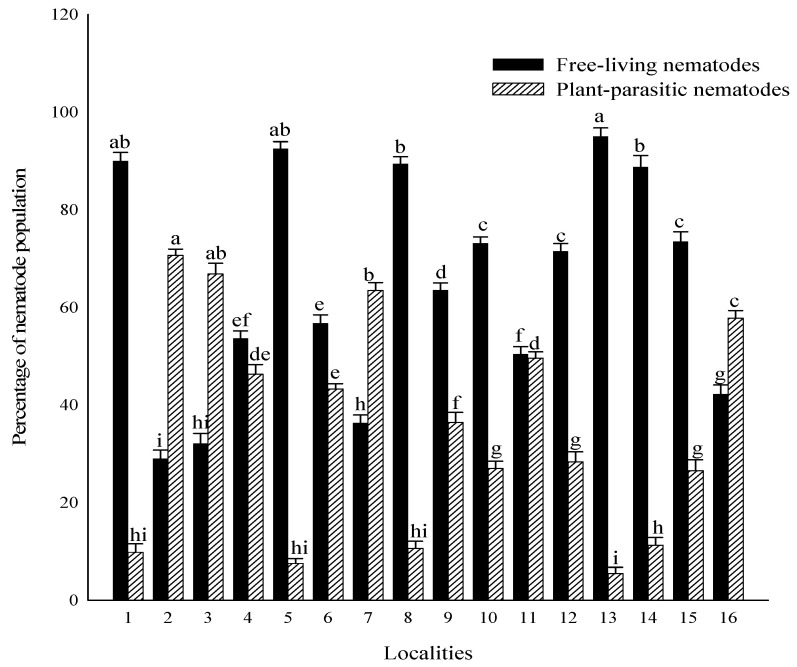
Occurrence (%) of plant-parasitic and free-living soil nematodes in maize and other rotational crops. The bars illustrate the mean and standard error. Different letters on the bars indicate significantly different values according to Duncan’s multiple range test at *p* > 0.05. Whereas; 1 (Bhalwal), 2 (Bumbi Zakhira Gashkori), 3 (Burj Jieway Khan), 4 (Chak 103 JB), 5 (Chak 22), 6 (Chak 35-2R/A), 7 (Chak Takht Hazara), 8 (Dera G. Umro Khan), 9 (Koat Maan Singh), 10 (Islampur), 11 (Mazaharabad), 12 (Noorpur), 13 (Pull Bagar), 14 (Shamsabad), 15 (Sheikhupura), 16 (123 EB Pakpattan Canal).

The overall proportion of plant-parasitic nematodes was 35% out of 210 soil samples of maize and other rotational crops, while free-living soil nematodes recovered 65% (Figure 2). Locality-wise, the highest percentage of plant-parasitic nematodes in Bumbi Zakhira Gashkori was 70.65%, while the highest percentage of free-living soil nematodes in Pakistan was 95.03%. Moreover, percentage occurrence of Free-living soil and plant-parasitic nematodes were 90% and 9.84% in Bhalwal; 29% and 70.65% in Bumbi Zakhira Gashkori; 33.13% and 66.82% in Burj Jieway khan; 53.13% and 46.29% in Chak 103 JB; 92.46% and 7.52% in Chak 22; 56.75% and 43.26% in Chak 35-2R/A; 36.32% and 63.45% in Chak Takht Hazara; 89.38% and 10.62% in Dera General Umro Khan; 63.54% and 36.41% in Islampur; 73.12% and 26.56% in Koat Maan Singh; 50.41% and 49.57% in Mazaharabad; 71.49% and 28.45% in Noorpur; 95.03% and 5.45% in Pull Bagar; 88.72% and 11.24% in Shamsabad; 73.47% and 26.52% in Sheikhupura and 42.24% and 57.75% in 123 EB Canal Pakpattan respectively (Figure 3).

### 3.2. Occurrence (%) of Most Frequently Encountered Plant-Parasitic Nematodes from Different Localities

**Figure 4:**
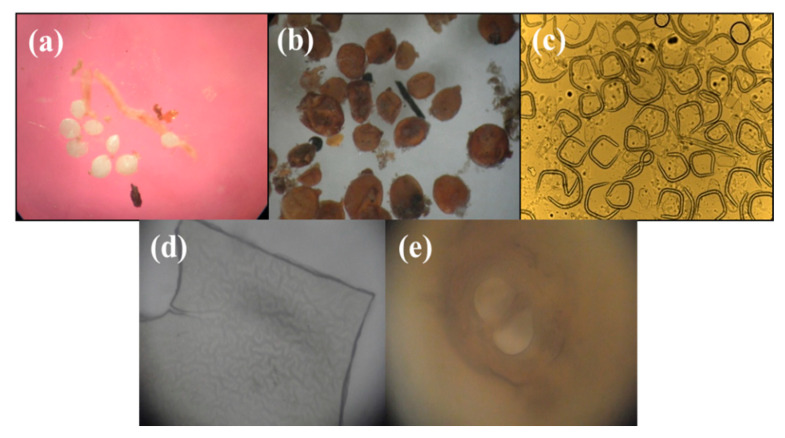
Different stages of maize cyst nematode (*Heterodera zeae*). (**a**) Young Females. (**b**) Mature cyst. (**c**) Juveniles. (**d**) Body pattern. (**e**) Cone.

Soil samples of maize and other crops, those cultivated in sequence with the maize fields, showed that the three important genera of plant-parasitic nematodes, *Heterodera, Tylenchorhynchus* and *Helicotylenchus,* were more prevalent and in a higher density than other plant-parasitic nematodes. Widespread occurrence (%) of plant-parasitic nematodes was followed, *Heterodera zeae*, with the highest occurrence (55.71%) followed by *Tylenchorhynchus elegans* (33.33%) and *Helicotylenchus certus* (24.76%). *Heterodera zeae* was more prevalent than other plant-parasitic nematodes in maize plantations and other crops cultivated in sequence with the maize fields (Figure 4). It is a major pest of maize. It was found in the active form; it was observed in a condition when it was attached to the roots. However, their percentage occurrence varied at each surveyed site (Table 1).

### 3.3. Community Analysis of Plant-Parasitic and Free-Living Soil Nematodes

Community analysis of plant-parasitic nematodes and free-living soil nematodes showed high variability in population densities and frequencies of nematodes in different localities. However, during the studies, some nematodes were more frequently encountered in surveyed sites. Locality wise, some parameters of the community structure of free-living soil nematodes, and plant-parasitic nematodes of maize plantation and other crops, were cultivated in sequence with the maize fields at Punjab, Pakistan. These are given in Table 2, Table 3, Table 4, Table 5, Table 6, Table 7, Table 8, Table 9, Table 10, Table 11, Table 12, Table 13, Table 14, Table 15, Table 16 and Table 17. Overall, the *Heterodera zeae* was a more frequently encountered species that is a major pest of maize. The nematode community structure of each locality contained parameters like; Absolute frequency (*AF*%), Relative Frequency (*RF*%), Relative density (*RD*%), and Prominence value (*PV*).

### 3.4. Nematodes Species Encountered during the Present Study

*Aphelenchoides besseyi* (Christie, 1942)

*Aphelenchus avenae* (Bastian, 1865)

*Bitylenchus brevilineatus* (Williams, 1960) Siddiqi, 1986

*Cephalobus nanus* (de Man, 1884)

*Ditylenchus clarus* (Thorne and Malek, 1968)

*Discolaimus texanus* (Cobb, 1913)

*Filenchus maqbooli* n.sp. (Aatika, Nasira and Shahina, 2017)

*Helicotylenchus certus* (Eroshenko and Nguen Vu Tkhan, 1981)

*Helicotylenchus gulabi* (Jain, Siddiqui and Aruna Parihar, 2000)

*Helicotylenchus jasminii* (Jain, Siddiqui and Aruna Parihar, 2000)

*Heterodera zeae* (Koshy, Swarup and Sethi, 1971)

*Heterodera mothi* (Khan and Hussain, 1965)

*Hoplolaimus indicus* (Sher, 1968)

*Malenchus labiatus* (Maqbool and Shahina, 1985)

*Hemicriconemoides cocophillus* (Loos, 1949) Chitwood and Birchfield, 1957

*Pratylenchus goodeyi* (Sher and Allen, 1953)

*Psilenchus minor* (Siddiqi, 1963)

*Rhabdilis producta* (Schneider, 1866) Linstow, 1878

*Rotylenchulus reniformis* (Linford and Oliveria, 1940)

*Seinura oostenbrinki* (Hussain and Khan, 1967)

*Telotylenchus indicus* (Siddiqi, 1960)

*Tylenchorhynchus elegans* (Siddiqi, 1961)

*Tylenchorhynchus tritici* (Golden, Maqbool and Handoo, 1987)

*Xiphinema bergeri* (Luc, 1973)

### 3.5. Cluster Analysis of Nematodes Species in Maize and Other Rotational Crops

#### 3.5.1. Cluster Analysis of Localities Regarding Nematodes Species Encounter from Maize

**Figure 5:**
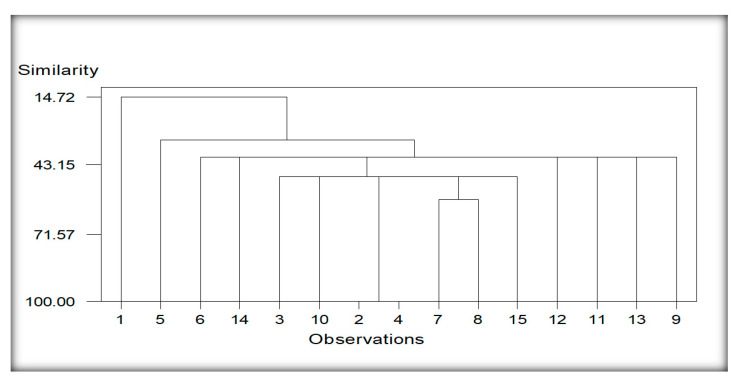
Cluster analysis of localities regarding nematodes species of maize. Whereas; 1 (Bhalwal), 2 (Bumbi Zakhira Gashkori), 3 (Burj Jieway Khan), 4 (Chak 103 JB), 5 (Chak 22), 6 (Chak 35-2R/A), 7 (Dera General Umro Khan), 8 (Islampur), 9 (Koat Maan Singh), 10 (Mazaharabad), 11 (Noorpur), 12 (Pull Bagar), 13 (Shamsabad), 14 (Sheikhupura), 15 (123 EB Pakpattan Canal).

The dendrogram generated due to 21 species of plant-parasitic and free-living nematodes depicts 7 cluster groups (Figure 5). Nematode species found from localities 1, 5, and 15 (Bhalwal, Chak 22, and 123 EB Pakpattan Canal) constituted separate and independent groups, whereas few common species from locality 6 and 14 (Chak 35-2R/A and Sheikhupura), 7 and 8 (Dera General Umro Khan and Islampur) and 12, 11, 13 and 9 (Pull Bagar, Noorpur, Shamsabad, and Koat Maan Singh) cluster together formulating independent groups but with variable similarities and linkage properties. Common nematode species of localities 3 and 10 (Burj Jieway khan and Mazaharabad) also clustered with nematode species found from the other two localities viz., 2, and 4 (Bumbi Zakhira Gashkori and Chak 103 JB) but with the weakest possible linkage and similarities ties.

#### 3.5.2. Cluster Analysis of Localities Regarding Nematodes Species Encounter from Potato

**Figure 6:**
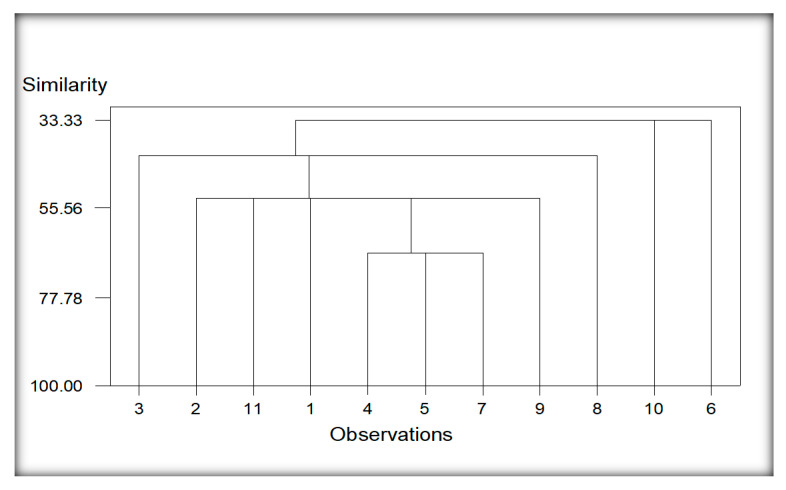
Cluster analysis of localities regarding nematodes species of potato. Whereas; 1 (Bumbi Zakhira Gashkori), 2 (Burj Jieway Khan), 3 (Chak 103 JB), 4 (Chak 35-2R/A), 5 (Dera General Umro Khan), 6 (Islampur), 7 (Mazaharabad), 8 (Noorpur), 9 (Pull Bagar), 10 (Sheikhupura), 11 (123 EB Pakpattan Canal).

Six distinct groups can readily be recognized in the dendrogram (Figure 6). Group 1, 4 and 5 developed as separate groups due to different nematode species recovered in 3 different localities viz., 3, 9 and 8 (Chak 103 JB, Pull Bagar and Noorpur, respectively). Group 2 comprises the common nematode species of three localities 2, 11 and 1 (Burj Jieway Khan, 123 EB Pakpattan Canal and Bumbi Zakhira Gashkori) with a comparatively higher similarities index compared to the other groups. Group 3 was formulated due to the highest similarities index among the common nematode species recovered from the 3 localities viz., 4, 5 and 7 (Chak 35-2R/A, Dera General Umro Khan and Mazaharabad). Group 6 comprises similar nematodes species found from the other two localities, viz., 10 and 6 (Sheikhupura and Islampur). The localities of group 2 have the *Cephalobus nanus, Discolaimus texanus, Helicotylenchus certus* and *Heterodera zeae* as the common nematode species, while *Aphelenchus avenae, Cephalobus nanus, Discolaimus texanus* and *Rhabditis producta* are similar nematodes isolated from three localities of group 3. Group 6 contained *Aphelenchus avenae, Cephalobus nanus, Discolaimus texanus, Filenchus maqbooli* n.sp., *Heterodera zeae, Pratylenchus goodeyi* and *Rhabditis producta* are common nematode species. *H. zeae* is present in 13 localities out of 15 localities. *Heterodera zeae* is the major pest of maize, and it was present in almost every field of maize, but its severity of infestation varies from locality to locality.

#### 3.5.3. Cluster Analysis of Localities Regarding Nematodes Species Encounter from Sorghum

**Figure 7:**
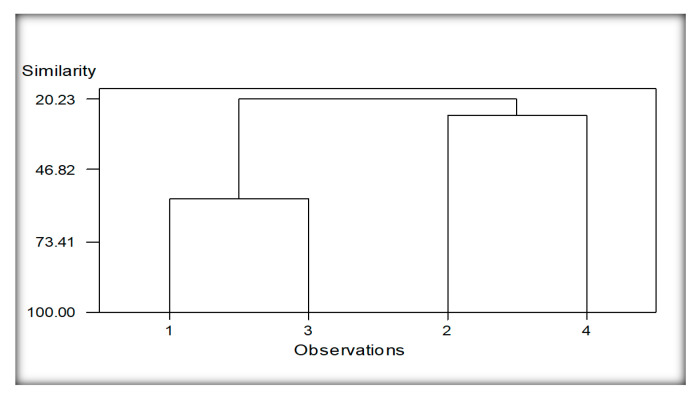
Cluster analysis of localities regarding nematodes species of Sorghum. Whereas; 1 (Burj Jieway Khan), 2 (Chak Takht Hazara), 3 (Islampur), 4 (Sheikhupura).

The dendrogram resulted from the clustering analysis of nematodes species of sorghum, in this case, given in Figure 7. Two main groups readily can be seen. Group 1 comprises the nematodes species isolated from soil samples collected from the rhizosphere around the roots of sorghum plants from two localities, viz., Burj Jieway Khan and Islampur (1 and 3). Group 2 was constructed due to the nematode species recovered from the other two localities of the same province, viz., Chak Takht Hazara and Sheikhupura (2 and 4). This group depicts its separate identity but comparatively lower linkage similarities among nematode species than group 1. It might be due to greater distance in the localities with variable climatic conditions and soil types. Common nematode species in groups 1 and 2 are *Cephalobus nanus, Discolaimus texanus, Heterodera zeae* and *Tylenchorhynchus elegans*.

#### 3.5.4. Cluster Analysis of Localities Regarding Nematodes Species Encounter from Wheat

**Figure 8:**
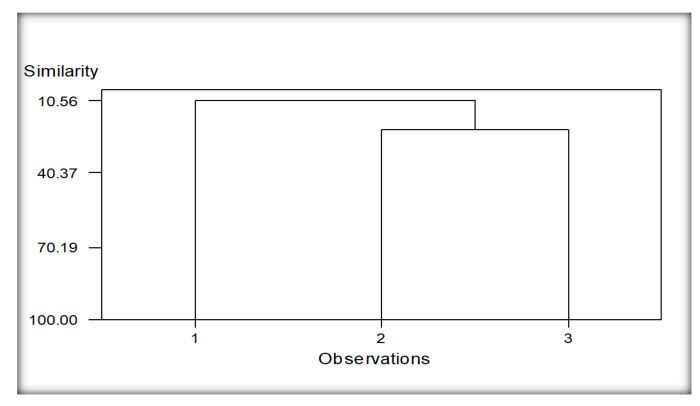
Cluster analysis of localities regarding nematodes species of wheat. Whereas; 1 (Burj Jieway Khan), 2 (Islampur), 3 (Sheikhupura).

The dendrogram developed from the data analysis showed two main clusters of wheat (Figure 8). Group 1 constituted due to nematodes species recovered from only the Burj Jieway Khan locality (1), while group 2 developed due to common nematodes species encountered from the rest of the two localities, viz., Islampur and Sheikhupura (2 and 3). The common species found in this group include *Aphelenchus avenae, Cephalobus nanus, Discolaimus texanus, Helicotylenchus certus* and *Rhabditis producta.*

#### 3.5.5. Cluster Analysis of Localities Regarding Nematodes Species Encounter from Tobacco

**Figure 9:**
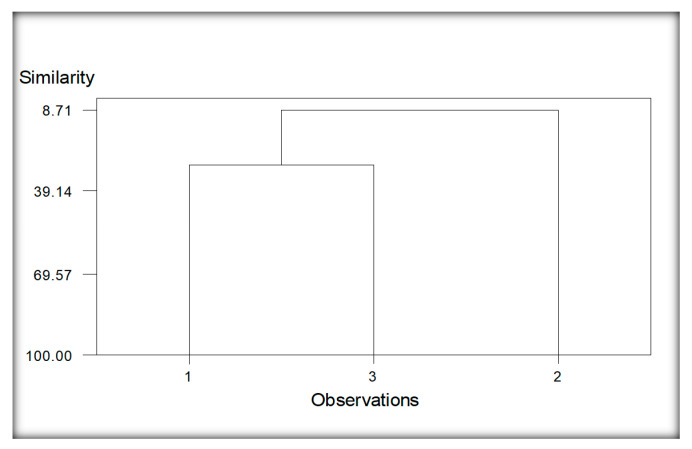
Cluster analysis of localities regarding nematodes species of tobacco. Whereas; 1 (Chak 103 JB), 2 (Chak Takht Hazara), 3 (Mazaharabad).

Analyzing different nematode species encountered from 3 localities generated 2 separate groups viz., 1 and 2 (Figure 9). Group 1 includes common nematode species recovered from two localities, Chak 103 JB and Mazaharabad (1 and 3) on Euclidian distance basis. Group 2 comprises nematode species recovered from only one locality, viz., Chak Takht Hazara (2). Eleven plant-parasitic and soil nematodes were encountered from tobacco plants during the studies, while the *Bitylenchus brevilineatus, Cephalobus nanus, Discolaimus texanus* and *Rhabditis producta* are common nematode species which present in all localities.

#### 3.5.6. Cluster Analysis of Localities Regarding Nematodes Species Encounter from Green Chilli

**Figure 10:**
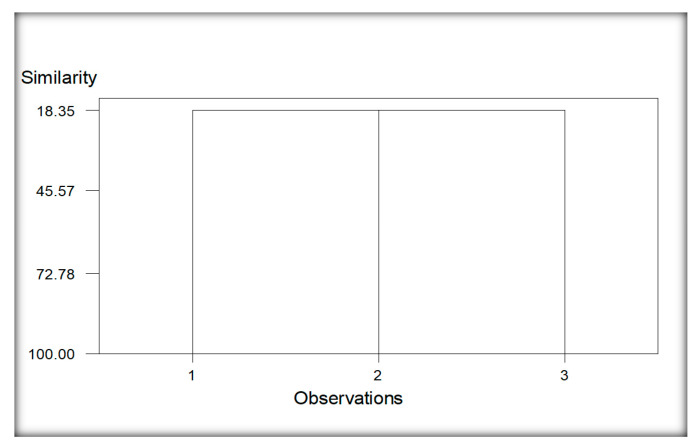
Cluster analysis of localities regarding nematodes species of chilli. Whereas; 1 (Chak 22), 2 (Chak Takht Hazara), 3 (Islampur).

A total of 10 species recovered from the soil around the roots of chilies plants from 3 different localities, viz., Chak 22, Chak Takht Hazara and Islampur (1, 2 and 3), were found clustered into the three distinct groups (Figure 10). Hence, all of the resultant groups contain different nematode species. The dissimilarities among nematodes might be because of different cropping patterns followed, water qualities, soil structure, and texture.

## 4. Discussion

Nematodes co-exist in different ecosystems, but their occurrence, density, and distribution depend largely on environmental and edaphic variables [50,51]. Nematodes can cause severe damage to maize. For community indicator analysis, nematodes can form the most relevant group because of more data about their taxonomy and feeding categories than other macro-fauna. Free-living nematodes are very effective and necessary for degrading organic materials and recycling soil nutrients. Free-living nematodes are essential for plant growth [52]. However, plant-parasitic nematodes inflict massive losses to the wide range of crops due to their yield and growth [53]. They can destroy crops through nurturing and by connecting with other microbes, which presents a major threat to crops, with an expected annual loss of USD 100–150 billion [54]. More than 120 species of nematode parasitize maize, and some of these species are known as primary pathogens [55,56,57]. Several major genera of plant-parasitic nematodes have been reported associated with maize: *Criconemella*, *Ditylenchus*, *Globodera*, *Haplolaimus*, *Helicotylenchus*, *Hemicriconemoides*, *Heterodera*, *Longidorus*, *Meloidogyne*, *Paratrichodorus*, *Pratylenchus*, *Radophulus*, *Rotylenchulus*, *Scutellonema*, *Trichodorus*, *Tylenchorhynchus* and *Xiphinema* [58].

However, the present findings confirm the results of previous surveys carried out in different countries by many researchers that worked on similar objectives viz., [59,60]. The present study revealed that 24 species were identified from maize and other rotational crops from 16 localities through Punjab, Pakistan. During the study, the most abundant plant-parasitic nematodes were, *Heterodera zeae*, with the highest incidence (55.71%), followed by *Tylenchorhynchus elegans* (33.33%) and *Helicotylenchus certus* (24.76%). However, the present findings confirm the results of previous surveys carried out by Mirsam, Muis and Nonci [60] that the *Heterodera* spp., *Tylenchorhynchus* spp. and *Helicotylenchus* spp. showed comparatively high frequency and density than for other trophic groups of nematodes in maize. Many researchers described that the cyst-infected maize exhibits extensively poor growth, is easily pulled out from the soil, is usually about half the size of healthy plants, forms patches in the field, discolors leaves turning yellow and sometimes turning reddish from tips that yield less grain than healthy plants [61,62,63,64,65]. During the present study, *H. zeae* was more prevalent in the maize field and other crops cultivated in nearby maize fields. Maize and other crops from nearby maize areas were invaded by several major nematodes that may cause numerous diseases.

During this study, the other rotational crops (such as potato, sorghum, wheat, tobacco and green chilli) were also studied because these crops were cultivated in sequence with the maize in the same field at the time of the survey. These crops have economic importance, but unfortunately, they suffer from several plant-parasitic nematodes. The previous studies reported that the cyst nematodes (*Heterodera* spp.), root-knot nematode (*Meloidogyne* spp.), stunt nematodes (*Tylenchorhynchus* and *Bitylenchus* spp.), spiral nematodes (*Tylenchorhynchus* spp.), lesion nematodes (*Pratylenchus* spp.), reniform nematodes (*Rotylenchulus* spp.), sheath nematodes (*Hemicriconemoides* spp.), rot nematode (*Ditylenchus* spp.), sting nematode (*Belonolaimus* spp.), stubby-root nematode (*Trichodorus* spp.), lance nematodes (*Hoplolaimus* spp.), foliar nematodes (*Aphelenchus* and *Aphelenchoides* spp.), ring nematodes (*Criconemoides* spp.) and dagger nematodes (*Xiphinema* spp.) are major plant-parasitic nematodes which are associated with potato, sorghum, wheat, tobacco and green chilli [22,66,67,68,69,70,71]. Moreover, during the present study, all of these nematodes were frequently present in all rotational crops. Migratory endoparasitic nematode, *Pratylenchus* reduced chlorophyll content, plant height, shoot and root weight also the destruction of the cortical parenchyma and epidermal tissues leading to severe root necrosis in maize [24,72,73]. During the present study, the *Pratylenchus* sp. was observed in maize and the other rotational crops of maize. Elhady, et al. [74] reported that the *Pratylenchus penetrans* was an endoparasite that invaded and migrated through roots as a juvenile or adult without becoming sedentary and escaped to the soil when conditions inside roots become adverse. Our previous study reported one new species *Filenchus maqbooli*, five new record species *Helicotylenchus certus*, *Helicotylenchus gulabi*, *Helicotylenchus jasminii*, *Pratylenchus goodeyi* and *Telotylenchus indicus* and two new host record *Tylenchorhynchus tritici* and *Malenchus labiatus* from maize [75].

The reniform nematode *Rotylenchulus* species commonly causes damage in tropical environments, and it causes 40–60% yield losses [24]. *Helicotylenchus* and *Tylenchus* were observed in maize and bean cropping systems [76]. However, *Tylenchus* spp. is not considered a major limiting factor in maize production even at high populations, probably because their economic importance has not been entirely ascertained [77]. Moreover, *Helicotylenchus* was the most frequently occurring genus in maize due to its cosmopolitan and polyphagous nature [78]. The high density of *Helicotylenchus* was correlated with reducing chlorophyll content, root mass and plant height in maize [79]. The migratory ectoparasites, namely, *Longidorus* and *Xiphinema* occurred in low abundance in this study. Our findings are consistent with Atandi, Haukeland, Kariuki, Coyne, Karanja, Musyoka, Fiaboe, Bautze and Adamtey [76], who found a low abundance of *Longidorus* and *Xiphinema* in maize and beans fields in Kenya. Our results correspond with Mokbel [80] recorded the following species associated with maize, namely *Criconemella* sp., and *Helicotylenchus* sp., *Heterodera* sp. grown in Abu-Arish governorate, Jizan province, Southwest, Saudi Arabia. Cluster analysis was also performed based on nematode communities in maize and other rotational crops. The resulting dendrograms showed a diversity level among the different localities of the maize and other rotational crops regarding the presence of nematodes species.

## 5. Conclusions

The present study reveals that 24 species of nematodes are present in maize and other rotational crops. Most of the species reported in this study are highly pathogenic, and thus their occurrence can pose severe threats to maize and other rotational crops and require the urgent attention of researchers. Special attention must be paid to the nematode attack hotspots found in the present case. The above will need to keep in mind the susceptibility level of these crops to the PPNs existing in the soils and ecosystems in which the plantations will need to be established. The outcomes of this research could be valuable in the future for identifying potential perturbations in the maize, such as agronomic methods, the application of biological control agents. In the future, this study will be a base for more research into the estimation of crop yield losses in maize and other crops cultivated with maize. This study underlined plant-parasitic and free-living nematodes variability and community structures as essential indicators for evaluating resilient approaches in maize growing systems. Further investigations can also concentrate on community rearrangements and relationships within species co-existence processes to establish methods for protecting or preserving diversity rather than removing the most pathogenic species.

## Figures and Tables

**Table 1:** Occurrence (%) of most frequently encountered plant-parasitic nematodes from different localities of Punjab, Pakistan.

		Positive Sample			Occurrence (%)		
Localities	Samples Collected	*H. Zeae*	*T. Elegans*	*H. Certus*	*H. Zeae*	*T. Elegans*	*H. Certus*
Bhalwal	27	12		4	44.44		14.81
Bumbi Zakhira Gashkori	11	9	4	1	81.81	36.30	9.09
Burj Jieway Khan	22	16	11	9	72.72	50	40.90
Chak 103 JB	7	6	2		85.71	28.57	
Chak 22	6						
Chak 35-2R/A	13	5			38.46		
Chak Takht Hazara	12	6	6	10	50	50	83.33
Dera G. Umro Khan	11	8	3		72.72	27.27	
Koat Maan Singh	7	4			57.14		
Islampur	31	17	25	7	54.83	80.64	22.58
Mazaharabad	6	3	3	2	50	50	33.33
Noorpur	17	8	7		57.05	41.17	
Pull Bagar	8		2	3		25	35.50
Shamsabad	6	3	2		50	33.33	
Sheikhupura	17	11	2	3	64.70	11.76	17.64
123 EB Pakpattan Canal	9	9	3	1	100	33.33	11.11
**Total:**	**210**	**117**	**70**	**40**	**55.71**	**33.33**	**19.04**

**Table 2:** Community structure of plant-parasitic and free-living soil nematodes in Bhalwal.

Nematodes	*AF* (%)	*RF* (%)	*RD* (%)	*PV*
*Aphelenchoides besseyi*	18.5 ± 1.05 ^g^	3.03 ± 0.09 ^d^	0.2 ± 0.09 ^f^	15.6 ± 0.98 ^i^
*Aphelenchus avenae*	74.1 ± 0.94 ^d^	12.1 ± 0.16 ^c^	2.4 ± 0.82 ^de^	366.7 ± 0.49 ^e^
*Cephalobus nanus*	92.6 ± 0.70 ^b^	15.2 ± 0.12 ^b^	6 ± 0.80 ^c^	1050 ± 1.90 ^c^
*Discolaimus texanus*	100 ± 1.70 ^a^	16.4 ± 0.46 ^a^	13.6 ± 2.16 ^b^	2462 ± 0.31 ^b^
*Ditylenchus clarus*	3.7 ± 1.45 ^j^	0.6 ± 0.09 ^e^	0.03 ± 0.01 ^f^	1 ± 0.55 ^k^
*Filenchus maqbooli* n.sp.	88.9 ± 2.65 ^c^	14.5 ± 0.95 ^b^	3.1 ± 0.95 ^d^	524.2 ± 1.09 ^d^
*Helicotylenchus certus*	14.8 ± 1.06 ^h^	2.4 ± 0.85 ^e^	1.6 ± 0.8 ^def^	112 ± 1.20 ^h^
*Heterodera zeae*	44.4 ± 1.25 ^f^	7.3 ± 0.10 ^d^	0.9 ± 0.33 ^ef^	117.8 ± 0.11 ^g^
*Rhabditis producta*	100 ± 1.50 ^a^	16.6 ± 1.27 ^a^	70.4 ± 1.06 ^a^	12,704 ± 0.99 ^a^
*Rotylenchulus reniformis*	3.7 ± 0.35 ^j^	0.6 ± 0.15 ^e^	0.1 ± 0.02 ^f^	2 ± 0.23 ^jk^
*Seinura oostenbrinki*	7.4 ± 0.1 ^i^	1.2 ± 0.90 ^e^	0.1 ± 0.09 ^f^	2.8 ± 0.18 ^j^
*Tylenchorhynchus tritici*	62.9 ± 0.85 ^e^	0.1 ± 0.08 ^e^	1.5 ± 1.41 ^def^	218.5 ± 1.27 ^f^

The data expressed the mean ± standard deviation. The different letters within columns are significantly different according to Duncan’s multiple range test (*p* > 0.05).

**Table 3:** Community structure of plant-parasitic and free-living soil nematodes in Bumbi Zakhira Gashkori.

Nematodes	*AF* (%)	*RF* (%)	*RD* (%)	*PV*
*Aphelenchus avenae*	9.1 ± 0.20 ^e^	3.0 ± 1.35 ^e^	0.4 ± 0.18 ^e^	1 ± 0.23 ^e^
*Cephalobus nanus*	100 ± 0.39 ^a^	33.3 ± 0.96 ^a^	22.2 ± 0.56 ^b^	165.8 ± 0.98 ^b^
*Discolaimus texanus*	54.5 ± 0.53 ^c^	18.2 ± 1.05 ^c^	4.4 ± 0.43 ^c^	24.5 ± 0.51 ^c^
*Helicotylenchus certus*	9.1 ± 0.17 ^e^	3.0 ± 0.30 ^e^	0.4 ± 0.05 ^e^	1 ± 0.24 ^e^
*Heterodera zeae*	81.8 ± 0.94 ^b^	27.3 ± 0.42 ^b^	69.3 ± 1.30 ^a^	468 ± 0.59 ^a^
*Rhabditis producta*	36.4 ± 0.23 ^d^	12.1 ± 1.09 ^d^	2.7 ± 0.09 ^d^	12 ± 0.56 ^d^
*Rotylenchulus reniformis*	9.1 ± 0.39 ^e^	3.0 ± 1.01 ^e^	0.4 ± 0.01 ^e^	1 ± 0.92 ^e^

The data expressed the mean ± standard deviation. The different letters within columns are significantly different according to Duncan’s multiple range test (*p* > 0.05).

**Table 4:** Community structure of plant-parasitic and free-living soil nematodes in Burj Jieway Khan.

Nematodes	*AF* (%)	*RF* (%)	*RD* (%)	*PV*
*Aphelenchus avenae*	4.5 ± 0.14 ^h^	1.2 ± 0.26 ^g^	0.2 ± 0.01 ^f^	1 ± 0.04 ^h^
*Cephalobus nanus*	95.5 ± 1.08 ^a^	25.0 ± 0.96 ^a^	20.4 ± 0.09 ^b^	481.2 ± 1.07 ^b^
*Discolaimus texanus*	54.5 ± 1.30 ^c^	14.5 ± 0.66 ^c^	7.4 ± 0.21 ^c^	131.6 ± 0.81 ^c^
*Helicotylenchus certus*	40.9 ± 1.01 ^e^	10.8 ± 0.98 ^e^	5.4 ± 0.44 ^d^	84 ± 1.06 ^d^
*Heterodera zeae*	72.7 ± 0.79 ^b^	19.3 ± 0.21 ^b^	56.6 ± 0.12 ^a^	1168 ± 1.50 ^a^
*Hoplolaimus indicus*	4.5 ± 0.28 ^h^	1.2 ± 0.37 ^g^	0.2 ± 0.15 ^f^	1 ± 1.14 ^h^
*Pratylenchus goodeyi*	4.5 ± 1.59 ^h^	1.2 ± 0.92 ^g^	0.2 ± 0.07 ^f^	1 ± 0.81 ^h^
*Rhabdolimus* sp.	4.5 ± 0.62 ^h^	1.2 ± 0.14	0.2 ± 0.17 ^f^	1 ± 0.15 ^h^
*Rhabditis producta*	36.3 ± 1.86 ^f^	9.6 ± 0.94 ^f^	5.2 ± 0.09 ^d^	76.4 ± 0.69 ^e^
*Rotylenchulus reniformis*	9.1 ± 0.29 ^g^	2.4 ± 0.44 ^g^	0.4 ± 0.01	2.82 ± 1.04 ^g^
*Tylenchorhynchus elegans*	50 ± 1.55 ^d^	13.3 ± 0.67 ^d^	3.9 ± 0.52 ^e^	66.3 ± 0.10 ^f^

The data expressed the mean ± standard deviation. The different letters within columns are significantly different according to Duncan’s multiple range test (*p* > 0.05).

**Table 5:** Community structure of plant-parasitic and free-living soil nematodes in Chak 103 JB.

Nematodes	*AF* (%)	*RF* (%)	*RD* (%)	*PV*
*Aphelenchus avenae*	14.3 ± 0.75 ^e^	3.1 ± 0.24 ^d^	0.3 ± 0.07 ^f^	1 ± 0.13 ^g^
*Cephalobus nanus*	100 ± 0.99 ^a^	21.3 ± 1.07 ^a^	21.3 ± 0.52 ^c^	177.2 ± 1.03 ^c^
*Chronogaster* sp.	14.3 ± 0.52 ^e^	3.1 ± 0.27 ^d^	0.3 ± 0.05 ^f^	1 ± 0.08 ^g^
*Discolaimus texanus*	85.7 ± 1.13 ^b^	18.8 ± 0.29 ^b^	6.9 ± 0.57 ^d^	53.9 ± 0.63 ^d^
*Helicotylenchus gulabi*	14.3 ± 0.11 ^e^	3.1 ± 0.56 ^d^	0.3 ± 0.06 ^f^	1 ± 0.93 ^g^
*Heterodera zeae*	85.7 ± 1.14 ^b^	18.8 ± 0.94 ^b^	42.5 ± 1.34 ^a^	328.2 ± 0.86 ^a^
*Rhabditis producta*	85.7 ± 0.96 ^b^	18.8 ± 0.10 ^b^	25.1 ± 0.11 ^b^	193.5 ± 0.28 ^b^
*Telotylenchus indicus*	19.0 ± 1.01 ^d^	4.2 ± 0.02 ^d^	1.1 ± 0.81 ^ef^	6.7 ± 0.14 ^f^
*Tylenchorhynchus elegans*	38.1 ± 2.15 ^c^	8.3 ± 0.97 ^c^	2.1 ± 0.56 ^e^	13.3 ± 1.06 ^e^

The data expressed the mean ± standard deviation. The different letters within columns are significantly different according to Duncan’s multiple range test (*p* > 0.05).

**Table 6:** Community structure of plant-parasitic and free-living soil nematodes in Chak 22.

Nematodes	*AF* (%)	*RF* (%)	*RD* (%)	*PV*
*Cephalobus nanus*	100 ± 1.07 ^a^	31.6 ± 1.03 ^a^	62.4 ± 0.87 ^a^	142.1 ± 0.92 ^a^
*Discolaimus texanus*	50 ± 1.10 ^b^	15.8 ± 0.08 ^b^	12.9 ± 0.29 ^b^	20.8 ± 0.42 ^b^
*Malenchus labiatus*	16.7 ± 0.12 ^d^	5.3 ± 0.56 ^d^	1.1 ± 0.12 ^e^	1 ± 0.28 ^e^
*Psilenchus minor*	16.7 ± 0.46 ^d^	5.3 ± 0.07 ^d^	2.2 ± 0.24 ^d^	2 ± 0.23 ^d^
*Rhabdolimus* sp.	16.7 ± 1.01 ^d^	5.3 ± 0.44 ^d^	1.1 ± 0.16 ^e^	1 ± 0.10 ^e^
*Rhabitis producta*	50 ± 1.06 ^b^	15.8 ± 0.21 ^b^	12.9 ± 1.03 ^b^	20.8 ± 0.53 ^b^
*Tylenchorhynchus tritici*	33.3 ± 0.65 ^c^	10.5 ± 0.26 ^c^	4.3 ± 0.36 ^c^	5.7 ± 0.12 ^c^

The data expressed the mean ± standard deviation. The different letters within columns are significantly different according to Duncan’s multiple range test (*p* > 0.05).

**Table 7:** Community structure of plant-parasitic and free-living soil nematodes in Chak 35-2R/A.

Nematodes	*AF* (%)	*RF* (%)	*RD* (%)	*PV*
*Aphelenchus avenae*	23.1 ± 0.99 ^f^	6.7 ± 0.09 ^e^	2.1 ± 0.57 ^e^	12.1 ± 1.05 ^f^
*Cephalobus nanus*	92.3 ± 1.02 ^a^	26.7 ± 1.28 ^a^	34.7 ± 0.21 ^b^	391.4 ± 2.07 ^a^
*Discolaimus texanus*	84.6 ± 0.86 ^b^	24.4 ± 1.11 ^b^	15.9 ± 0.52 ^c^	175.7 ± 1.25 ^c^
*Helicotylenchus jasminii*	15.4 ± 0.99 ^g^	4.4 ± 0.99 ^f^	0.6 ± 0.32 ^g^	2.3 ± 0.24 ^h^
*Heterodera zeae*	28.5 ± 0.74 ^e^	11.1 ± 0.28 ^c^	37.4 ± 1.11 ^a^	272.8 ± 1.06 ^b^
*Hoplolaimus indicus*	7.7 ± 0.11 ^h^	2.2 ± 0.19 ^g^	0.3 ± 0.04 ^g^	1 ± 0.28 ^h^
*Rhabditis produta*	38.5 ± 0.94 ^c^	11.1 ± 0.23 ^c^	6.1 ± 1.09 ^d^	44.7 ± 2.15 ^d^
*Telotylenchus indicus*	15.4 ± 0.39 ^g^	4.4 ± 0.01 ^f^	0.9 ± 0.18 ^fg^	7.4 ± 0.10 ^g^
*Tylenchorhynchus tritici*	30.8 ± 0.84 ^d^	8.9 ± 0.46 ^d^	1.8 ± 0.39 ^ef^	14.7 ± 1.15 ^e^

The data expressed the mean ± standard deviation. The different letters within columns are significantly different according to Duncan’s multiple range test (*p* > 0.05).

**Table 8:** Community structure of plant-parasitic and free-living soil nematodes in Chak Takht Hazara.

Nematodes	*AF* (%)	*RF* (%)	*RD* (%)	*PV*
*Aphelenchoides besseyi*	8.3 ± 0.97 ^j^	1.5 ± 0.29 ^h^	0.3 ± 0.11 ^i^	1 ± 0.92 ^n^
*Aphelenchus avenae*	41.7 ± 0.39 ^e^	7.4 ± 0.07 ^e^	6.2 ± 0.03 ^d^	49.2 ± 0.63 ^f^
*Cephalobus nanus*	100 ± 0.93 ^a^	17.7 ± 1.45 ^a^	22.1 ± 0.32 ^b^	273.7 ± 1.2 ^b^
*Discolaimus texanus*	66.7 ± 1.25 ^c^	11.8 ± 0.17 ^c^	10.3 ± 0.53 ^c^	104.7 ± 1.81 ^c^
*Filenchus maqbooli* n.sp.	33.3 ± 0.87 ^g^	5.9 ± 0.74 ^f^	1.1 ± 0.08 ^fi^	8 ± 0.44 ^m^
*Helicotylenchus certus*	83.3 ± 0.47 ^b^	14.7 ± 1.45 ^b^	31.4 ± 0.92 ^a^	354.2 ± 0.95 ^a^
*Heterodera mothi*	12.5 ± 0.57 ^i^	2.21 ± 0.82 ^h^	3.7 ± 0.48 ^e^	32.5 ± 0.13 ^i^
*Heterodera zeae*	37.5 ± 0.95 ^f^	6.6 ± 0.47 ^ef^	11.2 ± 0.28 ^c^	97.4 ± 0.74 ^d^
*Hoplolaimus indicus*	33.3 ± 1.05 ^g^	5.9 ± 0.49 ^f^	1.1 ± 1.01 ^fi^	8 ± 1.53 ^m^
*Psilenchus minor*	8.3 ± 0.96 ^j^	1.5 ± 0.15 ^h^	0.3 ± 0.28 ^i^	1 ± 0.42 ^n^
*Pratylenchus goodeyi*	8.3 ± 0.94 ^j^	1.5 ± 0.28 ^h^	0.3 ± 0.22 ^i^	1 ± 0.95 ^n^
*Rhabdolimus* sp.	25 ± 2.15 ^h^	4.4 ± 1.17 ^g^	1.7 ± 0.13 ^f^	10.4 ± 1.01 ^l^
*Rhabditis producta*	41.7 ± 1.23 ^e^	7.4 ± 0.29 ^e^	1.9 ± 0.14 ^f^	15.7 ± 0.86 ^j^
*Tylenchorhynchus elegans*	55.6 ± 1.39 ^d^	9.8 ± 1.03 ^d^	6.8 ± 1.16 ^d^	68.4 ± 1.16 ^e^
*Xiphenima bergeri*	11.1 ± 0.97 ^i^	1.9 ± 0.48 ^h^	1.4 ± 0.21 ^f^	13.7 ± 0.79 ^k^

The data expressed the mean ± standard deviation. The different letters within columns are significantly different according to Duncan’s multiple range test (*p* > 0.05).

**Table 9:** Community structure of plant-parasitic and free-living soil nematodes in Dera General Umro Khan.

Nematodes	*AF* (%)	*RF* (%)	*RD* (%)	*PV*
*Aphelenchus avenae*	36.3 ± 1.06 ^c^	8.51 ± 1.18 ^c^	1.1 ± 0.11 ^f^	14 ± 1.11 ^f^
*Cephalobus nanus*	90.9 ± 2.02 ^a^	21.8 ± 2.03 ^a^	30.8 ± 1.06 ^b^	622.9 ± 2.04 ^b^
*Discolaimus texanus*	90.9 ± 0.43 ^a^	21.8 ± 0.91 ^a^	8.9 ± 0.21 ^c^	180.3 ± 0.86 ^c^
*Filenchus maqbooli* n.sp.	18.2 ± 0.99 ^e^	4.3 ± 0.18 ^e^	0.3 ± 0.26 ^f^	2.9 ± 0.80 ^g^
*Helicotylenchus gulabi*	18.2 ± 0.99 ^e^	4.3 ± 0.08 ^e^	0.3 ± 0.06 ^f^	2.9 ± 0.69 ^g^
*Heterodera zeae*	72.7 ± 0.85 ^b^	17.0 ± 0.49 ^b^	6.7 ± 0.47 ^d^	121.6 ± 0.99 ^d^
*Rhabditis producta*	72.7 ± 0.96 ^b^	17.0 ± 0.59 ^b^	49.7 ± 0.34 ^a^	899.4 ± 0.03 ^a^
*Tylenchorhynchus elegans*	27.3 ± 1.75 ^d^	6.4 ± 0.15 ^d^	2.2 ± 0.12 ^e^	24.3 ± 0.63 ^e^

The data expressed the mean ± standard deviation. The different letters within columns are significantly different according to Duncan’s multiple range test (*p* > 0.05).

**Table 10:** Community structure of plant-parasitic and free-living soil nematodes in Koat Maan Singh.

Nematodes	*AF* (%)	*RF* (%)	*RD* (%)	*PV*
*Aphelenchus avenae*	42.9 ± 0.96 ^e^	7.5 ± 1.21 ^e^	3.3 ± 0.37 ^e^	15.6 ± 0.91 ^g^
*Cephalobus nanus*	85.7 ± 0.86 ^b^	15 ± 0.48 ^b^	12.9 ± 0.98 ^b^	85.7 ± 1.49 ^b^
*Discolaimus texanus*	57.1 ± 0.99 ^d^	10 ± 0.75 ^d^	10.4 ± 0.64 ^c^	56 ± 1.38 ^d^
*Ditylenchus clarus*	42.9 ± 0.65 ^e^	7.5 ± 0.72 ^e^	1.5 ± 0.10 ^f^	6.9 ± 0.37 ^h^
*Filenchus maqbooli* n.sp.	14.3 ± 0.90 ^f^	2.5 ± 0.53 ^f^	0.4 ± 0.24 ^f^	1 ± 0.92 ^i^
*Heterodera zeae*	57.1 ± 0.99 ^d^	10 ± 0.96 ^d^	7.8 ± 0.57 ^d^	20 ± 0.17 ^f^
*Pratylenchus goodeyi*	42.9 ± 0.91 ^e^	7.5 ± 0.55 ^e^	1.1 ± 0.07 ^f^	5.2 ± 0.53 ^h^
*Rhabdolimus* sp.	57.1 ± 0.16 ^d^	10 ± 0.86 ^d^	8.5 ± 0.73 ^d^	46 ± 1.37 ^e^
*Rhabditis producta*	100 ± 0.81 ^a^	17.5 ± 0.49 ^a^	41.4 ± 1.23 ^a^	296.3 ± 1.09 ^a^
*Telotylenchus indicus*	71.4 ± 0.45 ^c^	12.5 ± 0.34 ^c^	12.6 ± 0.26 ^b^	76 ± 1.21 ^c^

The data expressed the mean ± standard deviation. The different letters within columns are significantly different according to Duncan’s multiple range test (*p* > 0.05).

**Table 11:** Community structure of plant-parasitic and free-living soil nematodes in Islampur.

Nematodes	*AF* (%)	*RF* (%)	*RD* (%)	*PV*
*Aphelenchus avenae*	41.9 ± 0.98 ^f^	7.9 ± 1.61 ^e^	2.3 ± 0.66 ^e^	118.9 ± 0.99 ^f^
*Cephalobus nanus*	100 ± 1.08 ^a^	18.8 ± 0.49 ^a^	33.9 ± 0.93 ^a^	2739 ± 0.56 ^a^
*Discolaimus texanus*	90.3 ± 0.51 ^b^	16.9 ± 0.04 ^b^	8.9 ± 0.75 ^d^	687.9 ± 0.96 ^e^
*Filenchus maqbooli* n.sp.	9.7 ± 0.54 ^i^	1.8 ± 0.11 ^gh^	0.2 ± 0.09 ^h^	5.2 ± 0.22 ^l^
*Helicotylenchus certus*	20.2 ± 0.48 ^g^	3.9 ± 0.64 ^f^	1.2 ± 0.26 ^fg^	53.9 ± 0.47 ^g^
*Helicotylenchus gulabi*	8.1 ± 0.09 ^i^	1.3 ± 0.35 ^gh^	0.4 ± 0.11 ^gh^	17.9 ± 0.49 ^i^
*Helicotylenchus jasminii*	4.0 ± 1.52 ^j^	0.8 ± 0.16 ^h^	0.2 ± 0.18 ^h^	10.3 ± 0.15 ^j^
*Heterodera zeae*	54.8 ± 0.88 ^e^	10.3 ± 1.02 ^d^	18.9 ± 0.60 ^b^	1129.7 ± 1.17 ^c^
*Hoplolaimus indicus*	12.9 ± 1.01 ^h^	2.4 ± 0.21 ^g^	0.6 ± 0.11 ^fgh^	18 ± 0.79 ^i^
*Pratylenchus goodeyi*	9.7 ± 0.85 ^i^	1.8 ± 0.55 ^gh^	0.3 ± 0.27 ^h^	6.9 ± 1.09 ^k^
*Rhabdolimus* sp.	9.7 ± 0.77 ^i^	1.8 ± 0.59 ^gh^	1.3 ± 0.13 ^f^	32.9 ± 0.51 ^h^
*Rhabditis producta*	87.1 ± 0.96 ^c^	16.4 ± 0.19 ^b^	19.4 ± 0.43 ^b^	1460.1 ± 1.06 ^b^
*Tylenchorhynchus elegans*	80.7 ± 1.16 ^d^	15.2 ± 0.11 ^c^	12.4 ± 0.57 ^c^	900 ± 0.97 ^d^

The data expressed the mean ± standard deviation. The different letter within columns are significantly different according to Duncan’s multiple range test (*p* > 0.05).

**Table 12:** Community structure of plant-parasitic and free-living soil nematodes in Mazaharabad.

Nematodes	*AF* (%)	*RF* (%)	*RD* (%)	*PV*
*Aphelenchus avenae*	33.3 ± 1.14 ^e^	6.3 ± 1.11 ^d^	1.3 ± 1.09 ^g^	4.2 ± 0.77 ^i^
*Bitylenchus brevilineatus*	16.7 ± 0.85 ^f^	3.1 ± 0.99 ^e^	2.9 ± 0.79 ^ef^	13.5 ± 1.32 ^g^
*Cephalobus nanus*	100 ± 1.41 ^a^	18.8 ± 0.93 ^a^	28.8 ± 0.93 ^a^	166.6 ± 1.36 ^a^
*Discolaimus texanus*	83.3 ± 0.95 ^b^	15.6 ± 1.26 ^b^	11.9 ± 0.99 ^c^	62.6 ± 0.85 ^d^
*Filenchus maqbooli* n.sp.	16.7 ± 0.41 ^f^	3.1 ± 0.79 ^e^	0.4 ± 0.12 ^g^	1 ± 0.48 ^j^
*Helicotylenchus certus*	41.7 ± 1.24 ^d^	7.8 ± 0.76 ^cd^	9.5 ± 0.52 ^d^	38.9 ± 1.05 ^f^
*Helicotylenchus jasminii*	8.3 ± 0.29 ^g^	1.6 ± 0.39 ^e^	1.9 ± 0.63 ^fg^	7.8 ± 0.65 ^h^
*Heterodera zeae*	50 ± 1.17 ^c^	9.4 ± 1.21 ^c^	21.2 ± 1.15 ^b^	86.6 ± 1.11 ^c^
*Pratylenchus goodeyi*	33.3 ± 1.15 ^e^	6.3 ± 0.97 ^d^	3.8 ± 1.08 ^e^	12.7 ± 0.96 ^g^
*Rhabdolimus* sp.	16.7 ± 0.77 ^f^	3.1 ± 0.92 ^e^	0.4 ± 0.43 ^g^	1 ± 0.17 ^j^
*Rhabditis producta*	83.3 ± 0.87 ^b^	15.6 ± 0.74 ^b^	9.3 ± 0.56 ^d^	49.2 ± 0.89 ^e^
*Tylenchorhynchus elegans*	50 ± 1.07 ^c^	9.4 ± 1.17 ^c^	8.6 ± 1.02 ^d^	90.5 ± 0.85 ^b^

The data expressed the mean ± standard deviation. The different letters within columns are significantly different according to Duncan’s multiple range test (*p* > 0.05).

**Table 13:** Community structure of plant-parasitic and free-living soil nematodes in Noorpur.

Nematodes	*AF* (%)	*RF* (%)	*RD* (%)	*PV*
*Aphelenchus avenae*	35.3 ± 1.01 ^e^	8 ± 1.05 ^e^	1.9 ± 1.01 ^ef^	22.0 ± 1.16 ^f^
*Cephalobus nanus*	88.2 ± 0.87 ^a^	20 ± 0.99 ^a^	26.0 ± 0.96 ^a^	476.3 ± 0.91 ^a^
*Discolaimus texanus*	88.2 ± 1.01 ^a^	20 ± 0.86 ^a^	19.9 ± 0.21 ^d^	364.1 ± 0.91 ^c^
*Ditylenchus clarus*	5.9 ± 0.98 ^h^	1.3 ± 0.45 ^h^	0.4 ± 0.37 ^h^	2 ± 0.61 ^hi^
*Hemicriconemoides cocophillus*	5.9 ± 1.07 ^h^	1.3 ± 0.44 ^h^	0.2 ± 0.17 ^h^	1 ± 0.67 ^i^
*Helicotylenchus jasminii*	17.7 ± 1.05 ^f^	4 ± 0.61 ^f^	0.8 ± 0.26 ^gh^	6.9 ± 0.63 ^g^
*Heterodera zeae*	47.1 ± 0.83 ^c^	10.7 ± 0.71 ^c^	21.1 ± 0.81 ^c^	282.8 ± 0.71 ^d^
*Hoplolaimus indicus*	5.9 ± 0.92 ^h^	1.3 ± 0.62 ^h^	1.5 ± 0.48 ^fg^	7 ± 0.85 ^g^
*Malenchus labiatus*	11.8 ± 0.63 ^g^	2.7 ± 0.71 ^g^	0.4 ± 0.09 ^h^	2.8 ± 0.26 ^h^
*Rhabdolimus* sp.	11.8 ± 0.71 ^g^	2.7 ± 0.74 ^g^	0.4 ± 0.39 ^h^	2.8 ± 0.82 ^h^
*Rhabditis producta*	82.4 ± 0.47 ^b^	18.7 ± 0.71 ^b^	24.7 ± 0.71 ^b^	437.7 ± 0.69 ^b^
*Tylenchorhynchus elegans*	41.2 ± 0.39 ^d^	9.3 ± 0.35 ^d^	2.5 ± 0.17 ^e^	31.7 ± 0.70 ^e^

The data expressed the mean ± standard deviation. The different letters within columns are significantly different according to Duncan’s multiple range test (*p* > 0.05).

**Table 14:** Community structure of plant-parasitic and free-living soil nematodes in Pull Bagar.

Nematodes	*AF* (%)	*RF* (%)	*RD* (%)	*PV*
*Cephalobus nanus*	87.5 ± 0.98 ^a^	25.9 ± 0.91 ^a^	16.1 ± 0.84 ^b^	82.0 ± 0.48 ^b^
*Discolaimus texanus*	87.5 ± 0.54 ^a^	25.9 ± 1.03 ^a^	13.5 ± 0.48 ^c^	68.8 ± 0.86 ^c^
*Helicotylenchus certus*	12.5 ± 0.37 ^c^	3.7 ± 0.64 ^c^	2.6 ± 0.49 ^d^	5 ± 0.61 ^d^
*Pratylenchus goodeyi*	12.5 ± 0.54 ^c^	3.7 ± 0.39 ^c^	0.8 ± 0.59 ^e^	1 ± 0.92 ^f^
*Rhabdolimus* sp.	12.5 ± 0.33 ^c^	3.7 ± 0.73 ^c^	0.8 ± 0.39 ^e^	1 ± 0.79 ^f^
*Rhabditis producta*	87.5 ± 0.53 ^a^	25.9 ± 0.93 ^a^	64.6 ± 0.59 ^a^	328.1 ± 0.15 ^a^
*Telotylenchus indicus*	12.5 ± 0.51 ^c^	3.7 ± 0.69 ^c^	0.7 ± 0.07 ^e^	2.3 ± 0.31 ^e^
*Tylenchorhynchus* sp.	25 ± 0.38 ^b^	7.4 ± 0.42 ^b^	1.4 ± 0.38 ^e^	4.6 ± 0.43 ^d^

The data expressed the mean ± standard deviation. The different letters within columns are significantly different according to Duncan’s multiple range test (*p* > 0.05).

**Table 15:** Community structure of plant-parasitic and free-living soil nematodes in Shamsabad.

Nematodes	*AF* (%)	*RF* (%)	*RD* (%)	*PV*
*Aphelenchus avenae*	16.7 ± 0.75 ^d^	4.6 ± 0.59 ^d^	0.4 ± 0.14 ^f^	1 ± 0.73 ^f^
*Cephalobus nanus*	33.3 ± 0.45 ^c^	9.1 ± 0.66 ^c^	2.3 ± 0.56 ^d^	8.5 ± 0.49 ^d^
*Ditylenchus clarus*	16.7 ± 0.73 ^d^	4.6 ± 0.57 ^d^	0.4 ± 0.28 ^f^	1 ± 0.81 ^f^
*Discolaimus texanus*	50 ± 0.84 ^b^	13.6 ± 0.75 ^b^	9.4 ± 0.52 ^b^	43.3 ± 0.46 ^b^
*Helicotylenchus gulabi*	50 ± 1.59 ^b^	13.6 ± 1.16 ^b^	1.1 ± 0.85 ^ef^	5.2 ± 0.53 ^e^
*Heterodera zeae*	50 ± 0.93 ^b^	13.6 ± 0.82 ^b^	6.4 ± 0.56 ^c^	29.4 ± 0.64 ^c^
*Malenchus labiatus*	50 ± 1.76 ^b^	13.6 ± 0.75 ^b^	1.1 ± 0.06 ^ef^	5.2 ± 0.98 ^e^
*Pratylenchus goodeyi*	16.7 ± 0.70 ^d^	4.6 ± 0.4 ^d^	0.4 ± 0.13 ^f^	1 ± 1.14 ^f^
*Rhabditis producta*	100 ± 0.96 ^a^	27.3 ± 0.88 ^a^	77.1 ± 0.89 ^a^	502.1 ± 0.95 ^a^
*Tylenchorhynchus elegans*	33.3 ± 0.55 ^c^	9.1 ± 0.77 ^c^	1.5 ± 0.48 ^de^	5.7 ± 0.63 ^e^

The data expressed the mean ± standard deviation. The different letters within columns are significantly different according to Duncan’s multiple range test (*p* > 0.05).

**Table 16:** Community structure of plant-parasitic and free-living soil nematodes in Sheikhupura.

Nematodes	*AF* (%)	*RF* (%)	*RD* (%)	*PV*
*Aphelenchoides besseyi*	23.5 ± 0.48 ^g^	4.8 ± 0.80 ^ef^	0.3 ± 0.09 ^fg^	8 ± 0.64 ^i^
*Aphelenchus avenae*	29.4 ± 0.49 ^f^	5.9 ± 0.95 ^e^	0.4 ± 0.38 ^fg^	15.7 ± 0.54 ^h^
*Cephalobus nanus*	82.4 ± 1.41 ^c^	16.7 ± 1.16 ^b^	8.5 ± 0.55 ^b^	497.6 ± 0.63 ^c^
*Discolaimus texanus*	88.2 ± 0.74 ^b^	17.9 ± 0.45 ^ab^	9.5 ± 0.31 ^a^	580.6 ± 1.48 ^b^
*Filenchus maqbooli* n.sp.	41.2 ± 0.76 ^e^	8.3 ± 0.25 ^d^	0.6 ± 0.36 ^ef^	23.8 ± 0.73 ^g^
*Hemicriconemoides cocphillus*	5.9 ± 0.50 ^j^	1.2 ± 0.59 ^h^	0.1 ± 0.07 ^g^	1 ± 0.10 ^j^
*Helicotylenchus certus*	17.7 ± 0.71 ^h^	3.6 ± 0.27 ^fg^	2.2 ± 0.16 ^c^	58.9 ± 0.25 ^f^
*Heterodera zeae*	64.7 ± 0.51 ^d^	13.1 ± 0.25 ^c^	1.6 ± 0.28 ^d^	82.9 ± 0.50 ^e^
*Hoplolaimus indicus*	5.9 ± 0.56 ^j^	1.2 ± 0.31 ^h^	0.1 ± 0.09 ^g^	1 ± 0.23 ^j^
*Pratylenchus goodeyi*	11.8 ± 0.53 ^i^	2.4 ± 0.07 ^gh^	0.2 ± 0.09 ^fg^	4.2 ± 1.04 ^i^
*Rhabdolimus* sp.	5.9 ± 0.40 ^j^	1.2 ± 0.91 ^h^	0.1 ± 0.07 ^g^	1 ± 0.47 ^j^
*Rhabditis producta*	94.1 ± 1.07 ^a^	19.0 ± 0.19 ^a^	1.0 ± 0.23 ^e^	3476 ± 0.15 ^a^
*Tylenchorhynchus elegans*	11.8 ± 1.01 ^i^	2.4 ± 0.80 ^gh^	0.1 ± 0.07 ^g^	236.1 ± 0.98 ^d^
*Tylenchorhynchus tritici*	11.8 ± 1.03 ^i^	2.4 ± 1.23 ^gh^	0.1 ± 0.02 ^g^	236.1 ± 0.97 ^d^

The data expressed the mean ± standard deviation. The different letters within columns are significantly different according to Duncan’s multiple range test (*p* > 0.05).

**Table 17:** Community structure of plant-parasitic and free-living soil nematodes in 123 EB PakpattanCanal.

Nematodes	*AF* (%)	*RF* (%)	*RD* (%)	*PV*
*Aphelenchus avenae*	11.1 ± 0.96 ^e^	2.4 ± 0.43 ^e^	0.2 ± 0.17 ^d^	1 ± 0.17 ^f^
*Cephalobus nanus*	100 ± 1.01 ^a^	21.4 ± 1.43 ^a^	14.4 ± 1.59 ^b^	192 ± 1.59 ^c^
*Discolaimus texanus*	100 ± 2.06 ^a^	21.4 ± 2.08 ^a^	11.7 ± 2.25 ^c^	159 ± 2.25 ^d^
*Filenchus maqbooli* n.sp.	22.2 ± 0.72 ^d^	4.8 ± 0.69 ^d^	0.9 ± 0.91 ^d^	5.7 ± 0.91 ^e^
*Helicotylenchus certus*	11.1 ± 1.08 ^e^	2.4 ± 0.95 ^e^	0.4 ± 0.32 ^d^	2 ± 0.32 ^f^
*Heterodera zeae*	100 ± 1.52 ^a^	21.4 ± 1.55 ^a^	55.3 ± 0.94 ^a^	738 ± 0.94 ^a^
*Rhabditis producta*	88.9 ± 0.96 ^b^	19.1 ± 0.65 ^b^	16.2 ± 0.71 ^b^	203.6 ± 0.71 ^b^
*Tylenchorhynchus elegans*	33.3 ± 0.66 ^c^	7.1 ± 0.96 ^c^	0.9 ± 0.07 ^d^	6.9 ± 0.07 ^e^

The data expressed the mean ± standard deviation. The different letters within columns are significantly different according to Duncan’s multiple range test (*p* > 0.05).

## Data Availability

The authors confirm that the data supporting the findings of this study are available within the article.
